# On the origin of molecular oxygen in cometary comae

**DOI:** 10.1038/s41467-018-04972-5

**Published:** 2018-07-03

**Authors:** K. L. Heritier, K. Altwegg, J.-J. Berthelier, A. Beth, C. M. Carr, J. De Keyser, A. I. Eriksson, S. A. Fuselier, M. Galand, T. I. Gombosi, P. Henri, F. L. Johansson, H. Nilsson, M. Rubin, C. Simon Wedlund, M. G. G. T. Taylor, E Vigren

**Affiliations:** 1 Department of Physics, Imperial College London, Prince Consort Road, London, SW7 2AZ UK; 2 Physikalisches Institut, University of Bern, Sidlerstrasse 5, 3012 Bern, Switzerland; 3 LATMOS/IPSL-CNRS-UPMC-UVSQ, 94100 Saint-Maur, France; 4 BIRA-IASB, Royal Belgian Institute for Space Aeronomy, Ringlaan 3, Brussels, Belgium; 5 Swedish Institute of Space Physics, Ångström Laboratory, Lägerhyddsvägen 1, 752 37 Uppsala, Sweden; 6 Southwest Research Institute, P.O. Drawer 28510, San Antonio, TX 78228-0510 USA; 7 University of Texas at San Antonio, San Antonio, TX 78249 USA; 8 Department of Atmospheric, Oceanic and Space Sciences, University of Michigan, Ann Arbor, MI 48109 USA; 9 LPC2E, CNRS, 3 Avenue de la recherche scientifique, 45071 Orléans, France; 10 Swedish Institute of Space Physics, P.O. Box 812, 981 28 Kiruna, Sweden; 11 Department of Physics, University of Oslo, Sem Sælands vei 24, postbox 1048, 0317 Oslo, Norway; 12 European Space Agency, ESTEC, Keplerlaan 1, Noordwijk, 2200 AG The Netherlands

## Introduction

Molecular oxygen was detected^1^ in the coma of 67P by the on-board Rosetta Orbiter Spectrometer for Ion and Neutral Analysis (ROSINA)–Double Focusing Mass Spectrometer (DFMS)^2^. It had a high volume mixing of about 1–10%. Primordial O_2_ within the nucleus of comet 67P is compatible with the instrumental observations^1,3^. Yao and Giapis^4^ proposed an alternative mechanism. The Eley–Rideal (ER) reactions of energetic water-group ions with Si/Fe oxides, or other minerals present on the surface of the nucleus, can generate ${\mathrm{O}}_2^ -$. Yao and Giapis^4^ suggested that ${\mathrm{O}}_2^ -$ and energetic O_2_ (after photo-detachment) generated through ER reactions can be detected by ROSINA–DFMS and must be an important contributor of the O_2_ reported. In this correspondence paper, we do not disregard the fact that ER reactions can happen on the surface of the nucleus. However, we demonstrate that the amounts of ${\mathrm{O}}_2^ -$ produced through ER reactions cannot explain a significant fraction of the O_2_ detected by ROSINA–DFMS. In addition, we find that ${\mathrm{O}}_2^ -$ and energetic O_2_ generated through ER reactions could not be efficiently converted into the ROSINA–DFMS instrument and are therefore not responsible for the reported measurements.

To estimate the maximum amount of oxygen produced through ER reactions, we first consider the amount of ions, produced within the coma, that could impact the comet nucleus. Consistently with the multi-instrument analysis of Rosetta Plasma Consortium (RPC) and ROSINA sensors^5,6^, the number density of the expanding gas at a cometocentric distance *r* is^7^:

1 $$n(r) = \frac{Q}{{4\pi ur^2}}$$

where *Q* is the total outgassing rate and *u*, the outflow velocity. We assume a maximum global *Q* to estimate the maximum amount of ions produced at a given time. The global ionization frequency is taken to be *ν* = 10^−6^ s^−1^, which is an upper-bound based on measurements^8^. The production rate, d*P*, of ions in each spherical shell of thickness d*r* is constant:

2 $${\mathrm{d}}P = \nu n(r)4\pi r^2{\mathrm{d}}r = (\nu Q{\mathrm{/}}u){\mathrm{d}}r$$

Once produced, ions retain the velocity of their parent neutrals for several kilometers^5^. Eventually these ions are picked up by the solar wind and have been observed moving anti-sunward^9^. The impact rate Q^*^ onto the nucleus of radius *r*_0_ (2 km) for unidirectional motion can thus be estimated as:

3 $$Q^ \ast = {\int}_{r_0}^\infty \left( {\frac{{\pi r_0^2}}{{4\pi r^2}}} \right)\frac{{\nu {\kern 1pt} Q}}{u}{\mathrm{d}}r = \frac{{\nu {\kern 1pt} Q{\kern 1pt} r_0}}{{4u}}$$

**Fig. 1:**
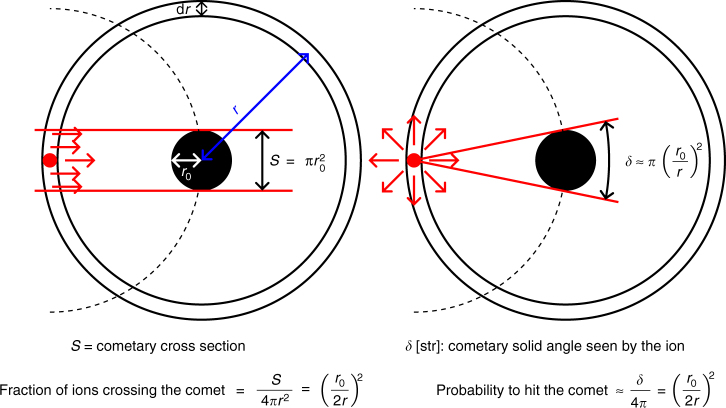
Schematic of the ion motion and their probability of returning to the nucleus. Shown for unidirectional motion (left side) and random motion (right side). In both case, the probability for an ion (red dot), created at *r*, to return to the nucleus is $\left( {\frac{{r_0}}{{2r}}} \right)^2$

This calculation also holds for random motion of ions (Fig. 1). It is somewhat simplistic but still provides an order of magnitude of the number of ions hitting the nucleus. Furthermore, even coupled with a uniform circular gyration induced by the magnetic field, the amount of ions hitting a spherical target would remain the same. The farther away an ion is, the less-likely it is to return to the target. In addition, the presence of ambipolar electric fields to maintain ion-electron neutrality may repel ions approaching the nucleus^10^.

Let us assume that every collision leads to an ER reaction with 100% yield. The production rate of ${\mathrm{O}}_2^ -$ through this process is therefore directly approximated by Q^*^. We assume a realistic (but slow, to obtain an upper bound) neutral outflow velocity of 500 m s^−1^. Q^*^ can be related to $Q_{{\mathrm{O}}_{\mathrm{2}}}$, the total production rate of O_2_ observed by ROSINA–DFMS, assuming low O_2_ mixing ratio (1%) for our upper-bound estimation^1^:

4 $$Q^ \ast = {Q_{{{\mathrm {O}}_2}}^{\mathrm {ER}}} \simeq 10^{ - 6}Q \simeq 10^{ - 4}{Q_{{{\mathrm {O}}_2}}} \ll {Q_{{{\mathrm {O}}_2}}}$$

The maximum fraction of ${\mathrm{O}}_2^ -$ anions produced through ER reactions, followed by photo-detachment—process which is associated with small cross sections^11^—is therefore insignificant with respect to the observed $Q_{{\mathrm O}_2}$^1^. Furthermore, extrapolating the lab samples^4^ as surrogates of the cometary surface is highly speculative given the nucleus porosity (30–65%^12^) and the presence of C and N bearing refractory species^13^ within the surface composition.

An alternative approach is to compute the energetic ion fluxes that could potentially strike the nucleus. Nilsson et al.^9^ computed 24-h average time series of the cometary energetic ion fluxes measured by RPC-Ion Composition Analyzer (ICA)^14^ over the 2-year mission. The maximum cometary energetic ion flux measured was about 10^13^ m^−2^ s^−1^. It leads to a total surface production rate $Q_{{\rm {O}}_{2}}^{ER}$ of ${\mathrm{O}}_{2}^ {-}$ of the order of 10^20^ s^−1^. It is still too low to reach volume mixing ratios of 1–10%: even for a low $Q_{\mathrm {O}_{2}}$ (1% mixing ratio of O_2_ and low *Q* of 10^26^ s^−1^)^1,7^, $Q_{\rm {O}_{2}}^{ER}$ is about $10^{ - 4}{Q_{{\mathrm {O}}_{2}}}$.

**Fig. 2:**
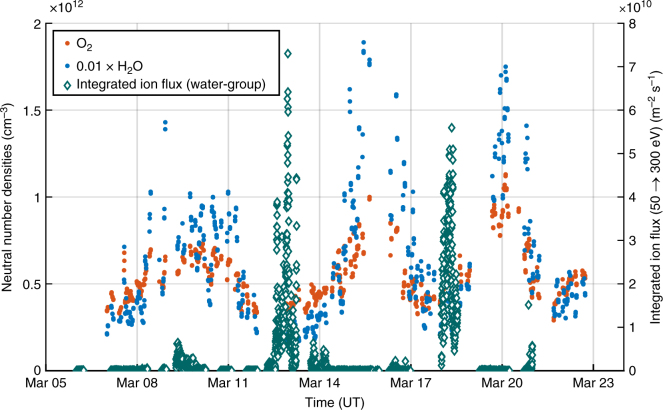
Time series of oxygen and water neutral number densities. Time series of the O_2_ (orange) and H_2_O (blue) neutral number densities (left *y*-axis) from March 6 to 23, 2016. The spacecraft was located at about 10 km from the nucleus. Note that the H_2_O neutral densities have been multiplied by 10^−2^ to fit on this scale. Time series of the total RPC–ICA energetic water-group ions (H_2_O^+^, H_3_O^+^, OH^+^) of cometary origin (green), corrected for the RPC–LAP spacecraft potential^15^ and integrated over the effective energy range of the Eley–Rideal reactions (50–300 eV)^4^ (right *y*-axis)

Figure 2 shows a time series of the energetic cometary water-group ion fluxes measured by RPC–ICA close to the nucleus from 5 to 23 March 2016. After correction for the negative spacecraft potential measured by the Rosetta dual Langmuir Probe (RPC–LAP)^15^, the flux is integrated over the 50–300 eV energies that are efficient to trigger the ER reactions^4^. The maximum value is 10^11^ m^−2^ s^−1^. The number densities of O_2_ and H_2_O derived from ROSINA–DFMS and ROSINA–COPS are over-plotted in Fig. 2. The fluctuations are essentially seasonal^16^ with high outgassing over the summer hemisphere and low outgassing over the winter hemisphere. There is a clear anti-correlation between O_2_ densities and the energetic ion fluxes, drivers of ER reactions. With a high outgassing, the flux is weakened by the time it reaches the nucleus due to ion-neutral collisions. The highest neutral densities of O_2_ are therefore not driven by energetic ions through ER reactions on the surface. In addition, O_2_ and H_2_O are strongly correlated^16^ (Fig. 2) and follow a *r*^−2^ Haser law^7^. This is incompatible with an extended source of O_2_ through ER reactions that could happen on dust grains or the spacecraft itself.

As for the instrumental detections, Yao and Giapis^4^ claim that ${\mathrm{O}}_2^ -$ and energetic O_2_ may hit the gold-coated surfaces of the ROSINA-DFMS and be efficiently converted into ${\mathrm{O}}_2^ +$. ROSINA–DFMS^2^ has two operation modes (neutral and ion) which differ by only the electrostatic bias and filament emission. In neutral mode, the ionization box is biased relative to spacecraft potential by +200 V to keep primarily cometary ions out and the filament emits electrons to ionize incoming neutrals. In ion mode, ROSINA–DFMS primarily measures thermal ions with no bias voltage on the ionization box and the filament is heated below the electron emission threshold (sub-emission). The angular acceptance of the electrostatic analyser is 5° and the energy acceptance is 1% of the nominal ion energy which depends on the mass measured. For mass 32 Da, this voltage is −1900 V: only species with energies below 19 eV pass the electrostatic analyser.

In neutral mode, ${\mathrm{O}}_2^ -$ anions with energies higher than the spacecraft potential (varying between 0 and −30 V^15^) would enter the instrument and be accelerated to about 200 eV. The impact of the gold surface to convert into ${\mathrm{O}}_2^ +$ would have to be accompanied by the loss of the 200 eV with minimal scattering to fit into the narrow angular acceptance angle. It is rather unlikely.

In the case of energetic O_2_, following the suggested conversion to ${\mathrm{O}}_2^ +$ via collisions with the gold surface, these ions, with energy below 19 eV and minimal angular scattering (<5°), could enter the sensor equally well in neutral and ion modes. As such, the peak height for mass 32 Da would be the same in both modes. In neutral high resolution mode, the peak was continuously strong, normally exceeding 10^4^ ions. However, in the ion mode, the signal was below detection limit for mass 32 Da in high resolution. This precludes the possibility that ${\mathrm{O}}_2^ -$ and energetic O_2_ are efficiently ionized in the DFMS sensor by hitting the surfaces of the instrument. These observations are much more consistent with a thermal neutral O_2_ population, partially ionized^8^. If the ER mechanism is in action, its contribution to the O_2_ peak in the neutral mode is very minor.

## Conclusions

We have used generous assumptions in terms of production rates and ion fluxes to assess the production of ${\mathrm{O}}_2^ -$ through ER reactions as a mechanism to explain Rosetta observations. Even with these assumptions, the amount of O_2_ produced is insignificant (by several orders of magnitude) with respect to what was detected by ROSINA–DFMS. There are not enough ions in the coma and the series of events required to trigger these processes are individually too rare. Furthermore, cometary ion fluxes are anti-correlated to the O_2_ densities observed by ROSINA. Finally, in terms of the instrument itself, there is little evidence of the production and detection of products ${\mathrm{O}}_2^ -$ and energetic O_2_ by ROSINA–DFMS. While ER reactions may occur, they cannot explain the amounts of O_2_ detected. Primordial O_2_^1,3^ remains compatible with the quantities and trends of molecular oxygen measured by ROSINA–DFMS, while other theories^17^ discuss other plausible sources.
